# Translation, cross-cultural adaptation and validity study of the “Play Nicely Program: The Healthy Discipline Handbook” for use in Brazil

**DOI:** 10.1590/0034-7167-2022-0281

**Published:** 2023-12-04

**Authors:** Janaina Recanello Begui, Seth Scholer, Naiara Barros Polita, Maria Aparecida Baggio, Maria de Fátima Garcia Lopes Merino, Adriana Valongo Zani, Rosângela Aparecida Pimenta

**Affiliations:** I Universidade Estadual do Norte do Paraná. Bandeirantes, Paraná, Brazil; II Vanderbilt University. Nashiville, Tenesse, United States of America; III Universidade Estadual de Londrina. Londrina, Paraná, Brazil; IV Universidade Estadual do Oeste do Paraná. Cascavel, Paraná, Brazil; V Universidade Estadual de Maringá. Maringá, Paraná, Brazil

**Keywords:** Validation Study, Translating, Child Abuse, Health Personnel, Health Promotion, Estudio de Validación, Traducción, Maltrato a los Niños, Salud Pública, Promoción de la Salud, Estudo de Validação, Tradução, Violência Infantil, Profissionais de Saúde, Promoção da Saúde

## Abstract

**Objective:**

to describe the translation, cross-cultural adaptation and validity process of the “Play Nicely Program: The Healthy Discipline Handbook” for use in Brazil.

**Methods:**

a methodological study that followed the translation, back-translation, expert committee assessment, and pre-test steps. The Content Validity Index (CVI) was calculated for both the judge population and the pre-test population. Four translators, seven expert judges in the field of child health and thirty participants in the pre-test, including parents, teachers and healthcare professionals, participated in the study.

**Results:**

in experts’ analysis (98.4%), a value of 100% of adequate assessments was obtained, and in the target population’s analysis (89.5%), there were 100% of adequate assessments. In both analyses, suggested adaptations were made.

**Conclusios:**

cross-cultural adaptation and content validity into Brazilian Portuguese of the “Play Nicely Program: The Healthy Discipline Handbook” were considered adequate for application in the target population.

## RESUMO

**Objetivo:**

desenvolver o processo de tradução, adaptação transcultural e validação do “*Play Nicely Program: The Healthy Discipline Handbook”* para uso no Brasil.

**Métodos:**

estudo metodológico, seguindo as etapas de tradução, retrotradução, avaliação do comitê de juízes e pré-teste. Foi calculado o Índice de Validade de Conteúdo (IVC) tanto para a população de juízes quanto para a população do pré-teste. População do estudo: quatro tradutores, sete juízes especialistas na área de saúde da criança e trinta participantes no pré-teste com pais, professores e profissionais da saúde.

**Resultados:**

na análise dos especialistas (98,4%), obteve-se valor de 100% de avaliações adequadas e, na análise da população-alvo (89,5%), totalizaram-se 100% de avaliações adequadas. Em ambas análises, foram realizadas adaptações sugeridas.

**Conclusão:**

a adaptação transcultural e a validação de conteúdo para o português do Brasil do “*Play Nicely Program: The Healthy Discipline Handbook*” foram consideradas adequadas para a aplicação na população-alvo.

## RESUMEN

**Objetivo:**

desarrollar el proceso de traducción, adaptación transcultural y validación del *“Play Nicely Program: The Healthy Discipline Handbook”* para uso en Brasil.

**Métodos:**

estudio metodológico, siguiendo las etapas de traducción, retrotraducción, evaluación por el comité de jueces y preprueba. Se calculó el Índice de Validez de Contenido (IVC) tanto para la población de jueces como para la población previa a la prueba. Población de estudio: cuatro traductores, siete jueces expertos en el área de salud infantil y treinta participantes en la preprueba con padres, docentes y profesionales de la salud.

**Resultados:**

en el análisis de los especialistas (98,4%), se obtuvo un valor de 100% de valoraciones adecuadas y, en el análisis de la población objetivo (89,5%), Se totalizó el 100% de las evaluaciones adecuadas. En ambos análisis, se realizaron adaptaciones sugeridas.

**Conclusiones:**

la adaptación transcultural y la validación de contenido para el portugués brasileño del *“Play Nicely Program: The Healthy Discipline Handbook”* se consideraron adecuadas para su aplicación en la población objetivo.

## INTRODUCTION

Child violence is a global public health concern and has had a significant impact on contemporary society, although it is a remote problem^(1)^. The consequences are numerous and can be short to long term, affecting children’s physical, psychological and emotional development^(2)^.

Approximately 25% of all children face some form of abuse or neglect during their lifetime. Any violation of a child by an adult, resulting in harm or injury, is considered child abuse^(3-4)^.

A study on child violence and its psychological consequences, carried out in Brazil, showed several long-term psychological disorders, such as depression, anxiety, post-traumatic stress disorder (PTSD), hyperactivity, attention deficit, emotional, affective, psychological, social sequelae and behavioral, requiring more studies on the subject in the country, in order to highlight the need for more effective actions in the protection and prevention of child violence^(5)^.

It is known that there is an intergenerational cycle of child abuse, i.e., those who were abused as children are more likely to abuse their own children^(6)^. Moreover, violence can be perpetrated throughout life as youth violence, intimate partner violence and elder abuse^(7)^. One of the seven strategies proposed to reduce child violence worldwide is to create ways to reduce physical or humiliating punishment and help parents understand the importance of positive, non-violent discipline with their children^(8)^.

Although Brazil has advanced in the production of public policies for childhood, such as the Brazilian National Policy for Comprehensive Child Health Care (PNAISC - *Política Nacional de Atenção Integral à Saúde da Criança*), which provides comprehensive care for children in situations of violence and a set of actions and strategies for its prevention^(9)^, not all children are able to enjoy its benefits^(10)^, either due to difficult access or the dismantling of the protection network^(11)^. Healthcare and education professionals are part of this network and thus they can contribute to applying the law to protect children who have their rights violated^(12)^.

Developed countries were pioneers in the development of programs for the primary prevention of violence against children, with research on promising interventions, mainly the United States of America, Canada and Western Europe^(13)^. However, research shows that children living in communities in poorest countries are more likely to experience abuse and neglect, and exposure to violence is more prevalent in impoverished or isolated neighborhoods and communities^(14)^.

Obstacles to implementing universal programs for child violence prevention include high cost, the need for training and long execution time, resulting, therefore, in the difficulty in population compliance and recruitment, becoming a challenge when incorporated into practice, especially in the primary care environment^(15)^. One of the strategic actions of PNAISC, in violence prevention, is to promote the articulation of intrasectoral and intersectoral actions by those who make up the Rights Guarantee System (SGD - *Sistema de Garantia de Direitos*), which implies support for implementing measures to combat violations of children’s rights, in which programs are included^(9)^.

The “Play Nicely Program: The Healthy Discipline Handbook” (http://playnicely.vueinnovations.com/) was developed with the aim of preventing child violence and mitigating toxic stress. It is a universal, brief intervention program and does not require prior training^(16)^. The program structure focuses on adverse childhood experiences (ACEs), in which child abuse is included^(17)^. It consists of a printed material that offers 20 discipline strategies that can be used with children from 1 to 10 years old, to deal with childhood aggression, based on the following question: suppose you see a child hitting another, what would you do? It has 39 pages, in language accessible to the public and can be used by parents, teachers and healthcare professionals^(18)^.

As for the knowledge gap, it is highlighted that studies on prevention programs related to child violence, especially cross-cultural adaptation, are scarce in the country. Thus, this study is justified by the need and importance of validating an instrument for Brazilian Portuguese capable of preventing child violence present in the population, contributing to changes in behavior and awareness of health, education and family professionals, and also to broaden the bases for research in this area.

## OBJECTIVE

To describe the translation, cross-cultural adaptation and validity process of the “Play Nicely Program: The Healthy Discipline Handbook” for use in Brazil.

## METHODS

### Ethical aspects

In order to carry out this study, permission for translating the material was requested by the author of the program, with a favorable opinion. Psychological care was provided if necessary due to the possibility of discomfort or embarrassment when analyzing the material. The development of the study complied with the national and international standards of ethics in research.

### Study design, period, and site

This is a methodological study with a quantitative approach, proposed by Beaton *et al*. (2000). The translation, back-translation, expert committee assessment and pre-test steps^(19-20)^ as well as the COnsensus-based Standards for the selection of health Measurement INstruments (COSMIN)^(21)^ criteria were followed. Data collection took place from March 2020 to September 2021. The study was carried out in a small municipality in northern Paraná in two municipal schools with Elementary School and three Basic Health Units (BHU).

### Population or sample; inclusion and exclusion criteria

The study population consisted of four translators, two translators participating in the first step (translation) and two in the back-translation step, seven evaluators. The target population consisted of 30 participants, 10 parents/caregivers of children aged 1 to 10 years, 10 kindergarten and elementary school teachers and 10 healthcare professionals from BHU. Inclusion criteria for the judges included being an expert in the field of child health, a teacher, having a master’s or PhD degree and having more than five years of job tenure. For the target population (parents/caregivers, healthcare and education professionals), inclusion criteria included caring for children aged 1 to 10 years, having children and/or students also in this age group; and exclusion criteria included not being literate (in the case of parents/caregivers) and having visual impairment.

### Study protocol

The first step consisted of translation of the original material into Portuguese by two translators fluent in English, whose mother tongue was the target language. One translator had training in the health area and was aware of the translation goals, while the other had no training in the health area, called a naive translator. It was intended that the latter would be able to detect a meaning different from the original identified by the first translator, as he is less influenced by an academic goal, offering a translation that reflected the language used by that population, often highlighting ambiguous meanings in the source material. The inclusion criteria for translators were having experience in English for at least five years, over 18 years old and being a native Brazilian.

With the translator’s version 1 (T1) and the translator’s version 2 (T2) in hand, analyzes of discrepancies between the versions were carried out by the researcher and translators and, after the analysis, a synthesis of these versions was created, called version T12. Subsequently, the material was back-translated (B) by two different translators from the first stage. With the versions of back-translator 1 (B1) and back-translator 2 (B2) in hand, analyzes were performed between the versions by the translators, with the aim of ensuring that the translated version reflected the same content as the original version^(17)^. After analysis, the synthesis of these versions was originated, called version B12. The inclusion criteria for these translators included being born in an English-speaking country, over 18 years old, living in Brazil for at least two years and being fluent in Portuguese.

The translators of both stages were selected from a database of translators, indicated by other researchers belonging to research groups that frequently carry out the process of cross-cultural adaptation. This version was then sent for analysis and evaluation by the author of the program, with no suggestions for changes.

The third step consisted of assessment of a committee of seven judges, experts in the field of child health and PhD holders. The judges answered a Likert-type scale from 1 to 4 assessing equivalences as follows: *semantic* - whose objective is to assess whether the words have the same meaning as well as grammatical difficulties in translation; *idiomatic* - in the case of colloquialisms or idiomatic expressions that are difficult to translate; *conceptual* - refers to the validity of explored concept and events experienced by people in the target culture, since items may be equivalent in semantic meaning, but not conceptually; *cultural -* daily life often varies in different countries and cultures, so a given task may simply not be experienced in the target culture, even if it is translatable, and adaptation is required^(19)^. The guide was sent through Google Forms, and was divided according to the chapter covered and subdivided into paragraphs, to facilitate analysis. If the score was less than 4, a comment or suggestion was requested. The experts had a period of 30 days for their assessment.

The last step consisted of carrying out the pre-final version with the target population, called pre-test, with 30 participants (10 parents/caregivers, 10 teachers and 10 healthcare professionals).

In elementary schools, parents and teachers were selected through contact with school principals. Then, the researcher was added to a school WhatsApp group so that an invitation to participate in the research was made. For those who agreed to participate, a face-to-face meeting was scheduled with the researcher so that they could sign the Informed Consent Form (ICF) and answer a sociodemographic questionnaire. Upon acceptance, parents and teachers received a copy of the material.

**Figure 1:**
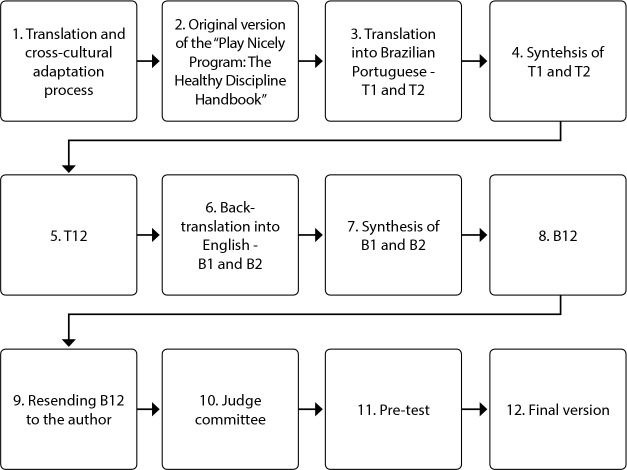
Synthesis of translation and cross-cultural adaptation of the “Play Nicely Program: The Healthy Discipline Handbook”

The contact with healthcare professionals was carried out through a nurse, BHU coordinator, who nominated the professionals available at that time. After accepting participation, a face-to-face meeting was scheduled with the researcher so that they could sign the ICF and respond to a sociodemographic characterization questionnaire; then they received a copy of the program. Participants were instructed on how to complete a Likert-type scale from 0 to 4 (0: I did not understand at all; 1: I understood a little; 2: I understood more or less; 3: I understood almost everything, but I had some doubts and 4: I understood perfectly and I have no doubts), also containing space available for suggestions or questions, if the score was less than 4. To facilitate handling and not forgetting what was read, the pages with the scale were added by section, that is, after each section or subject addressed, it was requested to fill it out. Figure 1 shows the translation and cross-cultural adaptation path.

### Analysis of results, and statistics

The collected data were structured in a Microsoft Excel^®^ spreadsheet database and later exported to IBM SPSS version 23 for statistical analysis. The Content Validity Index (CVI) was calculated for both the judge population and the pre-test population. The CVI was calculated by summing the agreement of items in the material, which reached a clarity scale with a score of “3” or “4” by all those who assessed it^(22-23)^. Items that received a score below “4” were reviewed, analyzed and adapted according to pertinent suggestions. The formula used to calculate each item’s CVI was obtained by the number of responses “3” or “4” divided by the total number of responses, which in the case of the experts, totaled 7, and the target population, 30 participants. The acceptable agreement index was at least 0.80 and preferably greater than 0.90^(22)^.

A total of 428 fields were analyzed in the expert committee step, and 38 fields, with the pre-test population. The difference in the number of fields was due to the way the sections were divided for assessment, and for expert committee assessment, as it is an extensive material, there was a need for greater fragmentation.

## RESULTS

The “Play Nicely Program: The Healthy Discipline Handbook” was adapted for Brazilian culture. The Brazilian version was entitled “*Brincar Legal: O Guia de Disciplina Saudável*”, and is structured in three chapters: 1) *Introdução*; 2) *Os cinco passos*; 3) *Quando procurar ajuda profissional*. In the subsection of chapter two, five recommendations of what to do in the face of acts of aggression are addressed: *Ensine seu filho a não ser uma vítima; Aprenda formas de agir frente a comportamentos desafiadores como a agressão, não ouvir, comportamento desrespeitoso, mentir e maus hábitos com 20 opções de disciplina; Diminua a exposição à violência e à mídia excessiva; Demonstre amor; Seja firme*.

From the first step of the translations (T1 and T2), their synthesis, T12, was obtained. For agreement between translations, adjustments were necessary by the researchers, which took place in two rounds. The translations had semantic discrepancies and were agreed upon so that the next step could be taken. In back-translation, B1 and B2 resulted in B12, the synthesis of B1 and B2. The translations in this step were very similar to the original version; when analyzed by the researchers, they highlighted some different terms, but without compromising the context. B12 was sent to the author of the program, with a favorable opinion, without changes. From the third and fourth step, the content validity process emerged.

The next step included expert analysis. The seven judges were female, nurses, holding a PhD, and teaching classes at an undergraduate nursing course in child health. Values obtained through CVI were: of the 428 analyzed fields, 7 (1.6%) obtained a value of 85.7% and 421 (98.4%) obtained a value of 100.0% in content validity. It was suggested by the judges to change the title of the word “*legal*” for “*saudável*” or “*gentil*”; however, the researchers chose to keep the word “*legal*”, as it better suits the material content. Regarding the change of the word “*livro*” for “*guia*” in terms of conceptual equivalence, the researchers decided to accept the suggestion, since the word “*guia*” aims at orientation, which corresponds better to the objectives of the material. Modified items are on Chart 1.

**Chart 1:** Modifications suggested by judges. Brazil, 2021

B12 version	Adaptation of judges’ suggestions
*“...têm maior risco de desenvolver problemas de saúde físicos ou mentais na vida futura”.*	*“...têm maior risco de desenvolver problemas de saúde físicos ou mentais no futuro”.*
*“Seria melhor se todas as crianças pudessem crescer em lares onde os pais usam opções saudáveis de disciplina”.*	*“Seria ideal se todas as crianças pudessem crescer em lares onde os pais usam opções saudáveis de disciplina”.*
*“Como agir frente a frente a comportamentos desafiadores...”*	*“A forma de agir frente a comportamentos desafiadores...”*
*“hábitos”*	*“maus hábitos”*
*“seja consistente”*	*“seja firme”*
*“...a amiga lhe sugere que ignore...”*	*“...a amiga lhe sugere ignorar...”*
*“...o que você pode fazer para agir...”*	*“...o que você pode fazer frente a..”*
*“...crianças mais pequenas...”*	*“...crianças menores...”*
*“Ensine seu filho a contar que foi agredido para um adulto responsável.”*	*“Ensine seu filho a contar para um adulto responsável que foi agredido”.*
*“...especialmente as mais pequenas...”*	*“especialmente as mais novas...”*
*“...a criança que foi agredida pode querer permanecer ali...”*	*“...a criança que foi agredida pode querer permanecer onde está...”*
*“considerando a mesma criança uma forma de agir pode funcionar bem em um dia, mas não no dia seguinte”.*	*“considerando a mesma criança o que funciona bem para uma criança em um dia, pode não funcionar bem no dia seguinte”.*
*“...vá à página correspondente e revise as 20 diferentes opções”.*	*“...vá à página seguinte e conheça 20 diferentes opções”.*
*“se você bater neles, eles aprenderão de você...”*	*“se você bater neles, eles aprenderão com você...”*
*“...os pastores usavam seus cajados para guiar as ovelhas e não bater nelas”.*	*“...os pastores usavam seus cajados para guiar as ovelhas e não para bater nelas”.*
*“Quando seu filho for grande o suficiente para entender isto, lhe pergunte sobre os sentimentos da pessoa que ele agrediu”.*	*“Quando seu filho tiver idade suficiente para compreender isto, lhe pergunte sobre os sentimentos da pessoa que ele agrediu”.*
*“...Perguntar a seu filho o que aconteceu...”,*	*“...Pergunte a seu filho o que aconteceu...”*
*“...não aceite os comentários do seu filho como desculpa para o comportamento agressivo”.*	*“...não aceite os comentários do seu filho para justificar o comportamento agressivo”.*
*“No final das contas, todos nos comportamos mal de vez em quando...”*	*“No final das contas, nós todos nos comportamos mal de vez em quando...”*
*“...e favorecerá um fluxo de comunicação entre você e seu filho que pode perdurar por anos futuros”.*	*“...e favorecerá um fluxo de comunicação entre você e seu filho que pode perdurar por muitos anos”.*
*“Você pode guiar o seu filho ao ensiná-lo como eles deveriam ter se comportado sem machucar ninguém.”*	*“Você pode guiar o seu filho ao ensiná-lo como ele deveria ter se comportado sem machucar ninguém.”*
*“Mentiras prejudiciais são quando as pessoas mentem para se safar de problemas ou obter coisas que não merecem”.*	*“Mentiras prejudiciais são quando as pessoas mentem para se livrar de problemas ou obter coisas que não merecem”.*
*“Você gostaria se eu desperdiçasse seu tempo toda vez que você quisesse ir a algum lugar?”*	*“Você gostaria que eu desperdiçasse seu tempo toda vez que você quisesse ir a algum lugar?*
*“Quanto mais você continuar praticando esse hábito, mais tempo levará para parar”.*	*“Quanto mais você continuar praticando esse hábito, mais tempo levará para que deixe de fazer”.*
*“...muitos atos violentos mostrados na mídia não trazem informações realísticas com o que aconteceu com as vítimas”.*	*“... muitos atos violentos mostrados na mídia não são acompanhados por informações realistas sobre as pessoas envolvidas”.*
*“... para crianças pequenas, principalmente as menores de dois anos...”*	*“...para crianças menores de 2 anos...”*
*“Guarde o seu celular e faça um contato visual com elas”.*	*“Guarde o seu celular e faça um bom contato visual com elas”.*
*“Resiliência é aquela força interna que permite às pessoas recuperar-se e vencer situações desafiadoras/difíceis”.*	*“Resiliência é aquela força interna que permite às pessoas recuperar-se e superar situações desafiadoras/difíceis”.*
*“Contudo, antes de abrir sua boca para dizer qual disciplina você vai usar, reflita consigo mesma...”*	*Contudo, antes de se comprometer em dizer qual disciplina você vai usar, reflita consigo mesma...”*
*“É melhor apoiar outros cuidadores...”*	*“É preciso apoiar outros cuidadores...”*

Of the adjusted changes, 99.7% were indicated as semantic equivalence and 0.3% were as idiomatic equivalence. The predominant sociodemographic characteristics in the target population were: 29 female participants (99%), and age ranged between 20 and 62 years, with 40% in the range of 30 to 40 years. Regarding education, most had completed high school (76.6%); and had two children (53.3%). Healthcare professionals’ and teachers’ job tenure ranged from 10 to 30 years (70%). From the target population’s assessment, the following results were obtained: of the 38 analyzed fields, 34 (89.5%) totaled 100% of adequate assessments, 3 (7.9%) totaled 96.7% of adequate assessments and 1 (2 .6%) obtained 93.3% of adequate assessments. Considering the results, we chose the changes in Chart 2.

**Chart 2:** Modifications suggested by the target population. Brazil, 2021

Pre-test version	Final version
*Disciplina “inapropriada”*	*Disciplina “inadequada”*
*Disciplina “apropriada”*	*Disciplina “adequada”*
*A “retaliação” geralmente leva ao agravamento da situação*	*“Revidar” geralmente leva ao agravamento da situação*
*Os homens fazem “progresso”*	*Os homens “evoluem”*

## DISCUSSION

Inappropriate discipline and childhood aggression are predictors for intergenerational violence maintenance, in addition to the increased risk of developing physical and mental health problems. In this regard, the program brings to the population healthy strategies for disciplining children, which can favor the interruption of this cycle^(18)^.

Parents’ negative reactions towards children, for not obeying inappropriate, ineffective and/or unclear commands given by them, may contribute to the initial activation of children’s problem behaviors. Furthermore, the fact that the program is universal, has a brief intervention and is offered within the scope of primary care favors the destigmatization of parents with identified needs. Long-term parenting intervention programs have been consistently linked to low compliance and related to conflicts in parenting priorities, schedules, and transportation problems^(24)^.

The translation, cross-cultural adaptation and validity process of the program took place systematically, following the stages of methodological framework of international credibility and criteria to ensure rigor and transparency^(25-26)^. The synthesis of the first validity step implied the observation of disparities between T1 and T2, and was highly successful by the researchers, being confirmed in the back-translation step. Thus, when sent to the author of the program for refinement of B12, and it did not receive indications for corrections or adaptations. Sending the program to the author showed good results in the previous steps.

Experts’ analysis pointed to suggestions that consolidated the material for use in the pre-test version. The fact that they work in teaching contributed positively to a more accurate correction rigor as it is part of their academic role. The content validity performed mostly showed corrections of semantic origin, preserving the meaning of the words contained in the original instrument^(19-20)^. In general, there were few notes considering material length and the number of fields to be assessed.

Idiomatic equivalence notes were essential, as in recommendation five, regarding the change of “*consistente*” for “*firme*”, which addresses the importance of parents/caregivers being firm when setting a rule for children and never ignoring aggressive behavior.

Establishing rules in raising children is implicit in the emotional needs common to all children, which is to establish realistic limits and not meet this need, which, combined with the non-fulfillment of other essential needs, can trigger negative repercussions throughout development child personality psychology^(27-28)^.

Being firm is different from harsh punishment. Power-imposed parenting, neglectful parenting, parenting methods of rejection, and child abuse are related to antisocial, aggressive, and violent behavior in adolescents^(29)^. On the other hand, studies show that the participatory style is related to higher levels of self-esteem and lower levels for authoritarian and neglectful styles^(30)^.

Another indication of change in idiomatic equivalence, pointed out by the experts, was the word “*hábitos*”, plus the word “*maus*”, resulting in the term “*maus hábitos*”. In addition to working on childhood aggression, the program has some recommendations for other challenging behaviors. These include thumb sucking, eating with the mouth open, lying, nail biting, curling the hair, and keeping people waiting too long. The bad habit of lying can present itself as an externalizing behavior. Problems of externalizing behavior in young children are common, have lasting negative impacts over time. When left unresolved, these behaviors are associated with family distress, parent-child relationship problems, and impaired social functioning. In the long term, early externalizing behaviors are associated with academic impairment, psychopathology, and significant economic costs^(31)^.

Regarding the target population, there was a prevalence of females, reflecting the mother as the main caregiver of their children. Although the scenario is evolving in the sense of changing a collective view on who is responsible for the role of caring and the father’s role is standing out^(32)^, society still has an intergenerational vision of women as leading roles in the care of children^(33)^.

Academic background and type and job tenure in the profession show the women’s search for different spaces, in addition to maintaining her role as caregiver^(32)^. In content validity, few suggestions emerged, all of which were semantic in origin, leading researchers to accept all suggested terms for a better understanding of the text. One of the words suggested was in the approach to discipline: replacing “*disciplina apropriada*” and “*disciplina inapropriada*” with “*disciplina adequada*” and “*disciplina inadequada*”. Inappropriate disciplines, such as physical punishment, are not permitted by law in several countries, including Brazil, which, in 2014, enacted Law 13010/2014, expressly prohibiting physical punishment of children and adolescents^(4,34)^; however, although the law is in force, the country has extremely high rates of physical violence against children.

Corporal punishment is defined as using physical force to make a child feel pain or discomfort, even if it is mild, including spanking^(35)^. Physical punishment does not predict improvements in children’s prosocial behavior or social competence over time, contributing to an increased risk of child abuse, i.e., in an environment where punishment is used as a way of educating, other types of abuse can evolve^(36)^.

Another change suggestion was the word “*retaliação*” for “*revidar*”. One of the recommendations that the program addresses is the importance of caregivers early teaching children not to be a victim of aggression and not to fight back if that happens. Aggressive behavior in young children is common, such as hitting, pushing, kicking, poking, pinching, biting, hair pulling, and picking up objects from other children. In this sense, the way caregivers will deal with this circumstance can mitigate or worsen the situation. The first model of social interaction that children have is their family and it is in this environment that their formation, concepts and belief in the world will be established^(37)^.

The program basis is the ACEs, which comprise five subtypes of child maltreatment, including physical violence. In recent years, the focus of research related to ACEs has shifted from delineating how they negatively affect health in adulthood to developing strategies for prevention in childhood. In this sense, the program stands out for addressing strategies of parenting practices that can contribute to interruption of violence intergenerational transmission^(38)^. Allied to this, the program applicability in realities such as Brazil’s can favor knowledge and practice on the subject.

### Study limitations

The absence of other healthcare professionals both in committee expert assessment and in pre-test due to difficulty in recruiting these professionals represents a limitation.

### Contributions to nursing, health, and public policies

The translation of the “Play Nicely Program: The Healthy Discipline Handbook” into Brazilian Portuguese can be used as a tool to help primary care healthcare professionals in assisting parents of children aged 1 to 10 years, educators in the classroom, and parents/caregivers in their homes. The program presents a set of actions for different populations that can be multiplied in the work environment and in the family/community life, which can favor, through the deepening of knowledge of healthy subjects, a more positive parenting regarding child education.

## CONCLUSION

The cross-cultural adaptation and validity of the “Play Nicely Program: The Healthy Discipline Handbook” was considered satisfactory for being easy to read and understand, demonstrated by the CVI of both experts and the target population, and can be useful for healthcare professionals, teachers and parents dealing with children from 1 to 10 years old. It can serve professionals as a tool in child care, teachers in the classroom, and parents/caregivers within their homes. Further studies on material applicability in the population for which it is intended are recommended to verify its usability in the country.
